# Serial mediating effects of childhood trauma and conduct behaviors on the impact of family history among patients with alcohol use disorder

**DOI:** 10.1038/s41598-024-57861-x

**Published:** 2024-03-26

**Authors:** Min Jeong Han, Shin Tae Kim, Chun Il Park, Syung Shick Hwang, Hae Won Kim, Jee In Kang, Se Joo Kim

**Affiliations:** 1 Institute of Behavioral Science in Medicine, Yonsei University College of Medicine, Seoul, South Korea; 2 Graduate School, Yonsei University College of Medicine, Seoul, South Korea; 3 Department of Psychiatry, CHA Bundang Medical Center, CHA University, Seongnam, South Korea; 4 Department of Medical Education, Institute of Behavioral Science in Medicine, Yonsei University College of Medicine, Seoul, South Korea; 5 Department of Psychiatry, Yonsei University College of Medicine, Seoul, South Korea

**Keywords:** Risk factors, Addiction

## Abstract

Family history (FH) of alcoholism increases the risk of alcohol use disorder (AUD); however, the contribution of childhood trauma (CT) in this respect remains unclear. This study investigated the relationship between FH and AUD-related clinical characteristics (social onset, antisocial tendency, and severity of problematic alcohol consumption) through the mediating effects of childhood trauma (CT) and conduct behaviors (CB) in a Korean male population with AUD. A total of 304 patients hospitalized for AUD at 16 psychiatric hospitals completed standardized questionnaires, including self-rated scales. Mediation analyses were performed using the SPSS macro PROCESS. Individuals with positive FH (133, 44%) had greater CT and CB and more severe AUD-related clinical characteristics than those without FH (171, 56%). In the present serial mediation model, FH had significant direct and indirect effects on AUD-related clinical characteristics through CT and CB. Indirect effects were 21.3% for social onset, 46.3%, antisocial tendency, and 37.9% for problematic drinking. FH directly contributed to AUD-related clinical characteristics, and CT and CB played mediating roles. This highlights the importance of careful intervention and surveillance of adverse childhood experiences and conduct disorder to prevent and mitigate alcohol-related problems in individuals with FH of AUD.

## Introduction

Family history (FH) of alcohol use disorder (AUD) in individuals is known to contribute to the development of AUD in subsequent generations, and numerous twin and adoptions studies showed that AUD is about 50% heritable^1–4^. In fact, having a positive family history (FH+) of AUD was found to be associated with more severe clinical characteristics, worse prognosis, and earlier onset of AUD than those without family history (FH−)^5–8^. Substantial evidence shows that FH+ individuals meet more diagnostic criteria for AUD and suffer from greater physical, inter-, and intrapersonal alcohol-related consequences^8–11^. Studies have found that FH is also closely related to behavioral and emotional problems that may predispose individuals to AUD, as FH+ individuals tend to have worse mood dysregulation and greater antisocial personality^12,13^. These findings demonstrate a close association between family history and AUD.

As family history is comprised of both genetic and environmental elements, both of these factors should be considered when studying the association between family history and AUD. Several genes associated with AUD have been identified, and many studies have revealed that genetic susceptibility is a crucial risk factor for AUD^2,14,15^. Furthermore, environmental factors such as childhood maltreatment and poor social support are also well-known risk factors for AUD development and severity^1,3,16^. Environmental factors can interact with genetic factors to increase the risk of AUD^1^. FH+ patients also reported greater early life adversity, which can independently increase the risk of AUD, regardless of FH^12,17^. This suggests a synergistic role for parental alcoholism in the pathogenesis and progression of AUD. Therefore, having biological parents with AUD is thought to expose genetically susceptible individuals to high-risk environments, which may increase the risk of AUD.

The relationship between FH and AUD can be affected by childhood experiences, particularly childhood trauma (CT). Early life trauma greatly increases the risk of substance abuse disorders through numerous mechanisms^1,18^. For instance, substance abuse can be a coping strategy for dealing with traumatic experiences, and dysfunctional family patterns may predispose an individual to early use of substances^19^. Such negative childhood experiences are more likely to be experienced by children with a FH of alcohol abuse^13^.

Furthermore, having parents with alcoholism can lead to the development of childhood behavioral problems, like antisocial behaviors, which are well-known risk factors for alcohol-related problems. Interestingly, conduct disorder is a predictor of regular alcohol use in early adolescence^20^. This can be explained by the role of impaired control during childhood, which can lead to earlier alcohol use, predisposing an individual to developing AUD in adulthood^20^. Such antisocial behaviors are more common in FH+ children, noting the importance of parental factors in the development of childhood personalities^21–23^. It can be thus said that childhood characteristics are crucial in the development of adult behaviors; and a FH of alcoholism has numerous genetic and environmental influences that can greatly increase the risk of AUD in individuals.

The current study aimed to explore the association between FH of alcoholism, negative childhood experiences such as CT and conduct behaviors (CB), clinical traits of AUD such as age at onset of social problems caused by drinking, antisocial tendency, and severity of problematic drinking. This study particularly investigated the mediating roles of CT and CB in the association between family history of alcoholism and age at social onset, severity of problematic drinking, and antisocial tendency using a serial multiple mediation model. We hypothesized that (1) the presence of a FH of alcoholism would be associated with certain AUD-related clinical characteristics, such as earlier onset, higher antisociality, and increased problematic drinking; (2) CT and CB would mediate the association between a FH of alcoholism and AUD-related clinical characteristics; and (3) CT and CB can act as serial mediators of the relationship between a FH of alcoholism and AUD-related clinical characteristics.

## Methods

### Participants

This study recruited 330 Korean male individuals with AUD from 16 psychiatric hospitals specializing in alcohol dependence with rehabilitation programs. All participants were inpatients who had been hospitalized primarily for alcohol detoxification rehabilitation. The inclusion criteria were as follows: (1) men aged between 20 and 70; (2) having a primary diagnosis of alcohol dependence according to the Diagnostic and Statistical Manual of Mental Disorders (DSM)-IV criteria; (3) being abstinent for at least one week prior to participation; and (4) having scored above the cut-off of eight points on the alcohol use disorders identification test (AUDIT), which is indicative of hazardous drinking^24,25^. The exclusion criteria were as follows: (1) having a major psychiatric disorder such as psychotic disorders, bipolar disorders, major depressive disorder, and anxiety disorder; (2) having substance dependence other than alcohol and tobacco use in the past six months based on the DSM-IV-TR criteria and clinician’s careful observation during hospitalization; (3) having scored less than 26 on the Mini Mental State examination—Korea version; (4) having mental retardation or organic mental disorder; and (5) having physical conditions preventing patients from complete self-reports and a structured, in-person interview^26–28^. All participants provided written informed consent before beginning the study, and the study protocol was approved by the Institutional Review Board of Severance Hospital. This study was performed in accordance with the approved guidelines.

### Measures

Participants completed a self-report questionnaire that included questions on date of birth, level of education, marital status, occupation, income, height, weight, and alcohol consumption. Of the 330, 26 did not provide appropriate answers to the questionnaire and thus were excluded from the analysis. The questionnaire included the following items:

1. Family history of alcoholism.

To assess family history of alcohol abuse, participants answered “yes/no” to whether they had any family members with problematic drinking and then specified who it was. An individual with a FH+ of alcoholism was defined as having at least one biological parent or sibling with a problematic drinking habit.

2. Social onset of AUD.

Social onset of AUD was defined as the earliest age at which participants experienced impairments in interpersonal, social, and occupational functioning due to harmful alcohol consumption. As part of the questionnaire, the patients were asked to specify the age at which they experienced social onset of AUD based on this definition. Two patients who did not provide answers were excluded from analysis.

3. The Personality Diagnostic Questionnaire—Version 4 + (PDQ-4 +) for antisocial tendency.

The PDQ-4 + is a 99-item yes/no tool used to screen for the 10 personality disorders of the DSM-IV axis II and the two personality disorders included in further studies that have shown reliability and validity in Korean populations^29,30^. Each “yes” response was counted as one point, and the sum of the points was used to evaluate the likelihood of having a personality disorder. Subscale scores were used to determine the presence of specific personality disorders. This study used a shortened 15-item version of the PDQ-4 + scale, which included eight questions assessing antisocial traits. The sum of the points for the eight items assessing antisocial traits was used to identify the extent of antisocial tendencies. Question 14 asked whether the participant had lied about many items in the questionnaire. 20 participants who answered “yes” to this question and four who did not answer were excluded from the analysis.

4. Modified Korean version of The Alcohol Use Disorders Identification Test (AUDIT-K) for severity of AUD.

The AUDIT is a 10-item scale used as a screening tool to assess the severity of hazardous drinking^25^. The Korean version of the AUDIT was used to measure AUD severity. Higher AUDIT-K scores correspond to a more problematic drinking pattern^31^.

5. Modified Korean version of the Parent–Child Conflict Tactics Scale (mPCCTS), Childhood Maltreatment Scale, and modified version of the Conflict Tactics Scale (mCTS) for childhood trauma.

CT was evaluated using the mean composites of childhood maltreatment, sexual abuse, and parental conflict. Childhood maltreatment was assessed using the mPCCTS, a 24-item scale based on the Parent–Child Conflict Tactics Scale developed by Straus et al^32,33^. It measures psychological and physical maltreatment, and neglect of children experienced before the age of 18. Sexual abuse was measured using the “sexual abuse” section of the Childhood Maltreatment Scale, comprising 10 items assessing exposure to sexual violence before the age of 18^34^. The scale includes eight items that evaluate minor assaults, such as verbal abuse and physical touch, and two items that evaluate severe assault, such as oral sex and sexual intercourse. Childhood parental conflict experienced before the age of 18 years was measured using the mCTS^35,36^. This 10-item scale comprises verbal violence, minor physical violence, and severe physical violence between parents. The mean composite score of childhood maltreatment, sexual abuse, and parental conflict was calculated by summing the z-scores of the individual scales and dividing them by three.

6. The Personality Diagnostic Questionnaire – Version 4+ (PDQ-4+) for conduct behaviors.

Of the eight questions assessing antisocial traits in the shortened version of the PDQ-4+ used in this study, the last question included 15 sub-questions that inquired about delinquent behavior that occurred before the age of 15. The sum of the points for this question assessed CB.

7. Modified Korean version of The Beck Depression Inventory.

The Beck Depression Inventory (BDI) is a 21-item self-report scale that measures symptoms of depression^37–39^. A modified Korean version of the BDI was used for this study, with each item comprised of three response choices ranging from absent to intense symptoms. The scores for each item were summed up to assess depression severity criteria.

8. Modified Korean version of the Beck Anxiety Inventory.

The Beck Anxiety Inventory (BAI) is a 21-item self-report scale that measures severity of anxiety^40,41^. Each item on the BAI is comprised of four response choices in order from least to most severe symptoms. The total points for each item were added to assess anxiety severity. The modified Korean version of this scale was used in this study.

### Statistical analysis

All statistical analyses were conducted using the IBM SPSS 26.0 software for Mac (SPSS Inc., Chicago, IL, USA). Descriptive statistical analyses were first performed to evaluate the baseline differences between those with a FH of problematic alcohol consumption and those with no FH. Correlations between the variables and baseline differences were also found. Using Preacher and Hayes’ method, serial multiple mediation models of CT and CB were used as mediators of the relationship between FH of problematic alcohol consumption and one of three outcomes (social onset of hazardous drinking, antisocial tendency, and severity of problematic drinking)^42^. Further, a bootstrapping analysis with 10,000 re-samples was conducted. Analysis was performed using the PROCESS function in SPSS, and Model 6 was used for the multiple serial mediation model. Standardized coefficients were reported. Mediation was considered significant if the 95% bias-corrected and accelerated CIs (lower limit (LL) and upper limit (UL)) for the indirect effect (IE) did not include 0^43^.

## Results

The demographic and clinical characteristics of the participants are presented in Table 1. Of the 330 patients recruited, two who did not provide answers on social onset, 20 who answered “yes” to question 14 on the PDQ-4+ , and four who did not answer to question 14 on the PDQ-4+ were excluded from the statistical analysis. A total of 133 (44%) FH+ participants and 171 (56%) FH- participants were included in the analysis. It was found that FH+ individuals experience more childhood maltreatment, parental conflict, and conduct behaviors. This group also was younger and had higher levels of depression. Furthermore, FH+ patients had earlier social onset, greater antisocial tendency, and higher severity of problematic drinking.

**Table 1:** Demographic and clinical characteristics of the participants (Values represent means ± standard deviations).

Characteristics	Family history ( +)	Family history (−)	*p*-value
	(n = 133)	(n = 171)	
Age, years	47.00 (8.27)	49.56 (7.29)	0.005*
Age at first drink, years	18.53 (5.37)	19.76 (7.27)	0.062
Duration of illness, years	18.17 (9.42)	16.19 (10.26)	0.76
Childhood trauma	0.13 (.80)	− 0.16 (0.64)	0.001*
Mean composite z-score	79.69 (79.65)	54.90 (77.22)	0.001*
mPCCTS			
mCTS	5.95 (7.37)	2.86 (3.96)	< 0.001*
Sexual abuse	4.65 (8.87)	3.80 (8.51)	0.395
Conduct behaviors	4.20 (3.33)	3.12 (2.74)	0.003*
BDI	19.95 (12.65)	17.14 (11.93)	0.048*
BAI	14.71 (11.38)	12.32 (11.39)	0.071
AUD-related clinical characteristics			
Social onset, years	28.83 (8.91)	33.42 (9.82)	<0.001*
Antisocial tendency	3.17 (1.59)	2.59 (1.38)	0.001*
AUDIT	27.92 (7.33)	25.11 (7.79)	0.001*

*p < 0.05.

Spearman’s correlations between CT, CB, social onset, antisocial tendency, and AUDIT score are shown in Table 2. There were significant correlations between all five variables. CT, CB, antisocial tendencies, and problematic drinking were positively correlated with each other, and social onset was negatively correlated with the other variables.

**Table 2:** Correlations among childhood trauma, conduct behaviors, social onset, antisocial tendency, and AUDIT score.

	1	2	3	4	5
1. Childhood trauma	1.00				
2. Conduct behaviors	− 0.426*	1.00			
3. Social onset	− 0.224*	− 0.227	1.00		
4. Antisocial tendency	0.256*	0.504*	− 0.193*	1.00	
5. AUDIT score	0.279*	0.296*	− 0.255*	0.429*	1.00

*p < 0.05.

**Figure 1:**
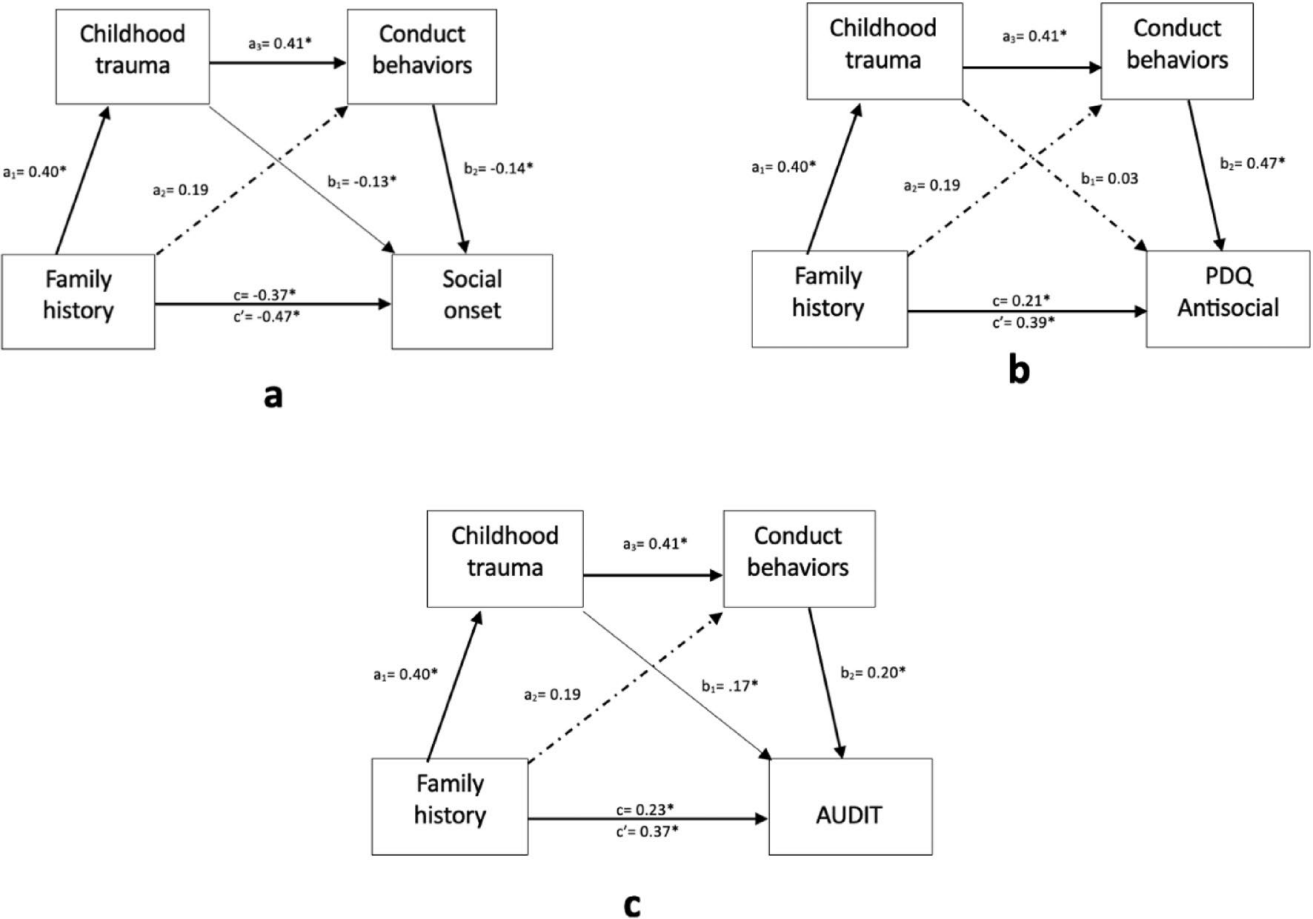
Path analysis linking family history of AUD with social onset (**a**), antisocial tendency (**b**), and severity of problematic drinking (**c**). a_1_ = direct effect of family history on childhood trauma; a_2_ = direct effect of family history on conduct behaviors; a_3_ = direct effect of childhood trauma on conduct behaviors; b_1_ = direct effect of childhood trauma on social onset (**a**), antisocial tendency (**b**), or severity of problematic alcohol consumption(**c**); b_2_ = direct effect of conduct behaviors social onset (**a**), antisocial tendency (**b**), or severity of problematic alcohol consumption (**c**); c’ = total effect of family history on social onset (**a**), antisocial tendency (**b**), or severity of problematic alcohol consumption (**c**); c = direct effect of family history on social onset (**a**), antisocial tendency (**b**), or problematic alcohol consumption (c). *p < 0.05*.* PDQ: Personality Diagnostic Questionnaire-4.

Serial multiple mediation models illustrating the relationships between family history and social onset, antisocial tendencies, and AUDIT scores in patients with AUD are presented in Fig. 1. Significant direct (SO: β = − 0.37, AS: β = 0.21, AD: β = 0.23) and total effects (SO: β = 0− 0.47, AS: β = 0.39, AD: β = 0.37) of FH on social onset, antisocial tendency, and severity of problematic drinking were found.

Table 3 summarizes these indirect effects. The indirect effect of FH on social onset through the path FH−> CT−> CB−> SO (β = − 0.0230, SE = 0.0123, 95% CI − 0.0513, − 0.0037) was significant. There was a significant indirect effect through CT (β = − 0.0510, SE = 0.0266, 95% CI − 0.1063, − 0.0030) and no significant effect through CB (β = − 0.0267, SE = 0.0193, 95% CI − 0.0715, 0.0027). The indirect effect of FH on antisocial tendency through the path FH−> CT−> CB−> AS (β = 0.0771, SE = 0.0244, 95% CI 0.0333, 0.1298) was significant. Contrastingly, the indirect effects through CT (β = 0.0138, SE = 0.0258, 95% CI − 0.0312, 0.0716) and through CB (β = 0.0895, SE = 0.0522, 95% CI − 0.0075, 0.1982) were not significant. The indirect effects of FH on severity of problematic drinking through the path FH−> CT−> CB−> AD (β = 0.0335, SE = 0.0140, 95% CI 0.0101, 0.0646) and through CT (β = 0.0680, SE = 0.0311, 95% CI 0.0147, 0.1356) were significant. However, the indirect effect through CB was not significant (β = 0.0386, SE = 0.0258, 95% CI − 0.0043, 0.0964).

**Table 3:** Standardized indirect effects for serial mediation models—results from 10,000 bootstrapping samples.

	Indirect effect	
	β (SE)	BC 95% CI (LL-UL)
SMM1: FH-> CT- > CB-> SO		
**FH-> CT-> SO**	− 0.0510 (0.0266)	(− 0.1063 to − 0.0030)*
FH-> CB-> SO	− 0.0267 (0.0193)	(− 0.0715 to 0.0027)
**FH-> CT-> CB-> SO**	− 0.0230 (0.0123)	(− 0.0513 to − 0.0037)*
**Total indirect effect**	− 0.1007 (0.0360)	(− 0.1783 to − 0.0366)*
SMM2: FH-> CT- > CB-> PDQ Antisocial		
FH-> CT-> AS	0.0138 (0.0258)	(− 0.0312 to 0.0716)
FH-> CB-> AS	0.0895 (0.0522)	(− 0.0075 to 0.1982)
**FH-> CT-> CB-> AS**	0.0771 (0.0244)	(0.0333 to 0.1298)*
**Total indirect effect**	0.1804 (0.0606)	(0.0674 to 0.3077)*
SMM3: FH-> CT-> CB-> AUDIT		
**FH-> CT-> AD**	0.0680 (0.0311)	(0.0147 to 0.1356)*
FH-> CB-> AD	0.0386 (0.0258)	(− 0.0043 to 0.0964)
**FH-> CT-> CB-> AD**	0.0335 (0.0140)	(0.0101 to 0.0646)*
**Total indirect effect**	0.1403 (0.0447)	(0.0596 to 0.2338)*

*p < 0.05. *LL* lower limit; *UL* upper limit, *FH* family history, *CT* childhood trauma, *CB* conduct behaviors, *AD* alcohol use disorder identification test, *AS* antisocial tendency, *SO* social onset; *SMM *serial mediation model

## Discussion

To the best of our knowledge, this study is the first to explore the relationship between FH and three AUD-related clinical characteristics (social onset, antisocial tendency, and severity of problematic alcohol consumption) through the serial mediating effects of CT and CB. Our findings showed that CT and CB significantly mediated this relationship, suggesting that adverse childhood experiences and behavioral problems together contribute to the pathogenesis of AUD. Additionally, the direct effects of FH on AUD-related clinical characteristics were found to be significant. These findings support previous theories that gene-environment interactions contribute to the disease phenotype in FH + individuals^1,44,45^.

In the serial mediation models, CT and CB partially mediated the relationship between FH and AUD-related clinical characteristics. In the mediation model of FH on social onset, the total indirect effect was significant (β =− 0.1007) and accounted for 21.3% of the total effect. The indirect effect of FH via CT (β = − 0.051) and that of the serial mediation via CT and CB (β = − 0.023) accounted for 10.9% and 4.9% of the total effect, respectively. The total indirect effect of FH on antisocial tendency was also significant (β = 0.1804), accounting for 46.3% of the total effect, and antisocial tendency was indirectly affected by FH serially through CT and CB (β = 0.0771), which accounted for 19.8% of the total effect. FH also had a significant total indirect effect on the severity of problematic drinking (β = 0.1403), and this accounted for 37.9% of the total effect. The indirect effect of FH via CT (β = 0.068) and that of the serial mediation via CT and CB (β = 0.0335) accounted for 18.4% and 9.1% of the total effect on severity of problematic drinking, respectively. These findings suggest that a FH is likely to predispose a child to experiencing trauma during childhood, which can trigger the development of CB. The cumulative effects of such stressors could lead to an earlier onset of social problems caused by drinking, greater antisocial tendency in adulthood, and more problematic consumption of alcohol. These findings corroborate previous studies showing that FH is a risk factor for both AUD development and severity^2–4,10^, and the results support our hypothesis that these effects are mediated by CT and CB.

Notably, we found a significant direct effect of FH on AUD-related clinical characteristics, which was greater than the indirect effects of CT and CB (78.7% vs. 21.3% for social onset, 53.8% vs. 46.3% for antisocial tendency, and 62.2% vs. 37.9% for severity of problematic drinking). These findings suggest that other environmental factors not included in this study may contribute to AUD pathogenesis and progression and that genetic aspects of FH may heavily influence AUD. In Korean men, lower educational status, lower service occupation, greater depressive symptoms, and being divorced or bereaved are associated with heavier drinking^46,47^. These mediators were not included in our analysis, which may account for the weaker indirect effects of FH.

Previous studies on childhood risk factors for AUD in FH + individuals have focused on adverse childhood experiences such as physical, sexual, or emotional abuse since they are known to be prevalent in families with a parental history of alcoholism. Our study not only considered CT, but also examined the additional effects of childhood misconduct on AUD. Pathways that included both mediators had significant indirect effects in all three models, suggesting that a causal effect could exist between them. A longitudinal study previously found that childhood maltreatment increased the risk of developing conduct disorder, which could be applicable to patients with CB^48^. Another study explained that early life stress leads to impulsive decision-making in children, a hallmark of both CB and substance abuse, further supporting the hypothesis made in this study^49^. Greater stress induced by multiple traumas during childhood can lead to various harmful outcomes, including the development of CB, thus predisposing already high-risk populations to develop worse clinical outcomes of AUD.

Interestingly, as shown in the Fig. 1, while three models showed significant serial mediating effects of CT and CB between FH and outcomes, no significant direct effect of FH on CB was found, suggesting a mediating role of CT between FH and CB. This reflects that in FH + AUD patients, CT is a critical risk factor for the development of CB. Previous studies have found CT to be a risk factor for the development of externalizing behaviors in adolescence, and CT is also associated with increased risk of alcoholism^1,12,50^. Children who are exposed to trauma develop impaired executive functioning, leading to problems with impulsivity control, and such traits have been shown to predict increased adolescent drinking and alcohol-related problems^51,52^. These findings implicate need for routine monitoring and surveillance for childhood abuse and neglect in individuals with a FH of AUD to reduce the risks of alcohol-related problems.

This study has several limitations. First, the cross-sectional design of this study limits the conclusions that can be drawn regarding the causal relationships among the variables. For instance, CT may not have occurred before CB, and antisocial tendencies may have been a precursor rather than an outcome of AUD. Second, only CT and CB were included as mediators in our study, which may not completely explain the relationship between FH and AUD-related clinical characteristics, as other mediators may be involved in the pathogenesis of AUD. Third, since the data were collected using a self-administered questionnaire, there is a possibility that the respondents did not answer accurately. In this study, the participants may not have recalled their FH accurately or answered correctly about their symptoms, which would have led to incorrect relationships between the mediator and outcome variables. Fourth, since the present study included only male patients with severe AUD requiring hospitalization, the results may not be applicable to females or those with mild-to-moderate cases of AUD. In addition, since we excluded patients with major depressive disorder and anxiety disorder, which often co-occur with AUD, to reduce heterogeneity of our sample, our findings may not be applicable to some AUD patients with diverse clinical levels of comorbid internalizing or externalizing disorders. Considering the presence of various comorbid psychopathologies in AUD, further research using a larger and a more diverse pool of participants is required to confirm the present findings.

## Conclusion

The results of this study indicate that FH directly and indirectly contributes to earlier social onset of alcohol-related problems, greater antisocial tendency, and more severe problematic drinking. The study found that CT and CB partially mediated the relationship between FH and AUD-related clinical characteristics. These findings highlight the importance of careful surveillance and an early identification of adverse childhood experiences and conduct problems to reduce the burden of childhood maltreatment and to prevent and mitigate alcohol-related problems in vulnerable individuals with a FH of alcoholism.

## Data Availability

Raw data are not publicly shared because the participants of this study did not give written consent for their data to be shared openly. However, the minimal datasets analyzed during the current study are available from the corresponding authors on reasonable request.
